# Bioresorbable scaffolds advances, challenges, and future directions

**DOI:** 10.1097/MS9.0000000000003424

**Published:** 2025-04-22

**Authors:** Muhammad Farhan, Gadeer Hasan, Abdulaziz Sobhi, Karim Yasser, Mohamad Humam, Mustafa Ali, Sara Younes, Muhammad Hashir Nazir, Mohammed Abdul Mateen, Gayatri Misra, Tirath Patel

**Affiliations:** a Department of Internal Medicine, Ajman University, College of Medicine, Ajman, United Arab Emirates; b Department of Internal Medicine, University of Sharjah, College of Medicine, Sharjah, United Arab Emirates; c Department of Internal Medicine, King Edward Medical University, Lahore, Pakistan; d Department of Internal Medicine, Shadan Institute of Medical Sciences, Hyderabad, India; e Department of Internal Medicine, American University of Antigua College of Medicine, Antigua and Barbuda; f Department of Internal Medicine, Trinity Medical Sciences University School of Medicine, Ratho Mill Kingstown, Saint Vincent and the Grenadines

**Keywords:** 3D printing, ABSORB IV, bioresorbable scaffolds, coronary artery disease, restenosis, stent thrombosis

## Abstract

Over the past few decades, researchers have attempted to overcome the disadvantages of metallic stents. This led to the birth of the first “Bioresorbable Scaffold” (BRS) model, the Absorb Bioresorbable Vascular Scaffold (BVS), in 1999 by Abbott. A stent that spontaneously resorbs shows a promising theoretical minimal risk of long-term stent thrombosis and omits the need for long-term antiplatelet therapy. However, only one year after its Food and Drug Administration (FDA) approval in 2016, it was voluntarily withdrawn owing to concerns regarding its safety because of the higher risk of target lesion failure. Although long-term follow-up data from the ABSORB IV trial were released in 2023 and showed a comparable long-term safety profile for BRS compared to conventional metallic stents, with only a slightly higher target lesion failure in the first three years, concerns remain regarding their use. Therefore, in this study, we discuss the different adverse events associated with different BRS models. We also discuss various approaches to optimizing the use of BRS, new BRS manufacturing techniques (e.g., 3D printing), and novel BRS models yet to be approved (e.g., DREAM 3G magnesium-based BRS). We suggest carefully selecting patients who could obtain the maximum benefit from BRS through the discussed selection criteria, which could reduce the risk of target lesion failure and BRS complications. Using newer technologies, such as 3D printing, also has excellent potential for making BRS more cost- and market-friendly, owing to their fast output and ability to form individually curated scaffolds.

**HIGHLIGHTS**

- Advances in bioresorbable scaffolds (BRS) enhance coronary artery disease management.
- Newer BRS models show reduced complications compared to earlier designs.
- 3D printing offers cost-effective and personalized BRS production potential.
- Patient and scaffold selection criteria are crucial to optimize BRS outcomes.
- Magnesium-based brs models improve strength and reduce thrombosis risk.

## Introduction

Coronary artery disease (CAD) is now reaching its highest recorded rates, both in incidence and mortality, with one out of every 20 persons above the age of 20 years developing CAD and one person dying from CAD every 33 s, based on the Centers for Disease Control and Prevention (CDC) statistics for 2023^[^1^]^. The increasing rates of CAD necessitate innovative therapeutic strategies to manage its complications effectively. One significant advancement in interventional cardiology has been the development of bioresorbable vascular scaffolds (BRS), which offer a potential alternative to conventional metallic drug-eluting stents (DES). Metallic DES have been associated with several complications, including stent thrombosis and late restenosis. A prominent concern is the chronic foreign body response elicited by the persistent presence of the stent within the artery. This long-term irritation potentially leads to neo-atherosclerosis and inflammation, which can compromise vascular health over time^[^2,3^]^.

Moreover, the physical presence of a metallic scaffold can inhibit vasomotion and adaptive remodeling of the arterial wall, factors that are critical for maintaining vascular function^[^4^]^. Studies have revealed that metallic stents can induce a pro-inflammatory state that persists long after the stent is implanted^[^5,6^]^. In contrast, BRS are designed to dissolve over time, theoretically reducing these risks and reestablishing normal vessel function post-absorption^[^7,8^]^.

The advantages of BRS over DES can be outlined in several key areas: biocompatibility, restenosis risk, and long-term complications. In terms of biocompatibility, BRS, made from polymeric materials, are designed to degrade naturally within the body, allowing for improved integration with the vessel wall and minimizing long-term inflammatory responses associated with permanent metallic implants^[^9,10^]^. Specifically, BRS significantly reduce the risk of late stent thrombosis compared to metal stents, which retain their structure indefinitely^[^11^]^. Clinical outcomes have shown that BRS can provide comparable efficacy regarding immediate post-procedure results while potentially improving long-term healing processes^[^12^]^. For example, a systematic review indicated that BRS yielded better outcomes in terms of patient recovery and vascular healing within the first 2 years postimplantation, enhancing the overall effectiveness compared to traditional metallic options^[^12^]^.

However, it’s essential to note that while BRS present a promising alternative, results have been mixed. Early trials showed enthusiasm for their potential; however, they faced skepticism due to subsequent reports of very late stent thrombosis, a phenomenon not sufficiently observed with DES^[^13,14^]^. Moreover, comprehensive follow-up studies are necessary to assess the long-term benefits and establish a clearer understanding of their performance over time in diverse patient populations, particularly in high-risk cases such as chronic total occlusions^[^15,16^]^.

In summary, while metallic DES have led to significant improvements in managing CAD, the limitations associated with their permanent nature underscore the need for alternatives like BRS. Following a trajectory toward enhanced biocompatibility and reduced long-term complications, BRS offer exciting prospects for the future of coronary interventions, although ongoing studies are essential to fully validate their long-term efficacy and safety.

## Earlier stent models

The first successful intervention to address CAD was coronary balloon angioplasty in the ‘70s. Although this technique was a medical revolution, it was associated with high failure rates owing to vascular elastic recoil, vessel remodeling, and proliferation^[^17^]^. This led to the development of the “prototype” bare metal stent (BMS) proposed in the 1980s^[^18^]^. Such stents overcame the obstruction but had a high risk of restenosis and stent failure. Research has aimed to overcome this drawback using drug-eluting stents (DES), which release sirolimus or paclitaxel, to overcome neointimal proliferation and reduce the risk of restenosis. Once again, the risk of chronic restenosis is reduced, but there is still a risk of acute stent thrombosis. Multiple modifications have been made to DES using thinner struts or biocompatible polymers to reduce the risk of stent thrombosis; however, the chronic presence of a vascular foreign body is still an ever-present issue that poses a risk of chronic complications^[^17,18^]^. Such complications include delayed vascular healing, vasomotor reactivity restoration, and side branch jailing^[^19^]^. Another possible drawback of permanent stents is that if coronary artery bypass graft surgery (CABG) is required later, a permanent stent could prevent such grafting^[^20^]^. This has led to research focusing on achieving a stent that achieves its short-term effect without being permanent; hence, the BRS concept was first proposed by Abbott in 1999^[^21^]^.

## Theoretical bioresorbable scaffold benefits

Since the development of Abbott’s BRS model in 1999, five BRS models have been assigned the CE mark according to European standards, and only the initial Absorb model proposed by Abbott was approved by the Food and Drug Administration (FDA) in the United States. BRS have become a new option for patients with cardiac diseases. Theoretically, they provided patients with benefits not observed in earlier stent models. Such benefits include^[^22,23^]^:

1. Lower rates of stent thrombosis, both due to drug elution during stent placement and the scaffold’s bioresorbable nature, prevent late stent thrombosis.
2. Return of standard vessel vasomotor capabilities after the resorption of the stent.
3. This reduced the need for long-term antiplatelet therapy to prevent stent thrombosis, thereby reducing the risk of bleeding.
4. Allowing for future percutaneous or surgical coronary interventions due to the lack of a permanent metallic stent.
5. With regular metallic stents, magnetic resonance imaging (MRI) is usually not feasible.
6. Drug elution was controlled by modifying the duration of biosorption. For example, early antiproliferative drug elution from the coating polymer is followed by late anti-inflammatory drug elution from the backbone polymer.
7. Eliminating psychological concerns of some patients regarding the presence of permanent implants within their bodies.

## The process of biosorption

Different scaffolds have different characteristics regardless of their effectiveness, resorption mechanism, and timeline^[^24^]^. More trials are being conducted to use different backbones and coating materials to improve the intrinsic attributes of the available BRS models. For example:

### Poly-L-lactic acid (PLLA, e.g., Absorb Bioresorbable Vascular Scaffold [BVS])

poly l-lactic acid, the primary polymer for most commercially approved BRS. It is a semicrystalline polymer with low tensile strength (60–70 Mpa) and elasticity (3.1–3.7 Gpa). The degradation process typically lasts for more than 24 months. The degradation process begins with the conversion of PLLA into lactate. Lactate is then converted to pyruvate, which is further converted to carbon dioxide water after entering the Krebs cycle, which is then excreted through renal excretion. Macrophages phagocytosed end-product particles smaller than 2 μm via a minimal inflammatory reaction. This leads to complete degradation of PLLA-based scaffolds^[^7,24^]^.

### Poly-d, l-lactic acid (PDLLA, e.g., ART pure)

Another similar polymer is PDLLA, which has similar characteristics to PLLA, although it has a slightly lower tensile strength (45-55 Mpa) and shorter degradation time (6-12 months)^[^24^]^.

### Magnesium alloy (e.G., Magmaris)

This scaffold backbone is being increasingly tested in different trials. The Magmaris scaffold is the only magnesium-based BRS approved in the market. Using magnesium as a scaffold platform (usually alongside other elements such as zinc, aluminum, and manganese) offers much higher tensile strength (220–330 Mpa) and elasticity (40–45 Gpa) as well as a lighter scaffold weight when compared to standard PLLA-based scaffolds. Its degradation rate was faster than that of PLLA. The degradation process for magnesium occurs through two phases: (1) the generation of magnesium hydroxide due to the interaction between the magnesium alloy and water, and (2) the conversion of magnesium hydroxide to calcium phosphate with a high water content. This biphasic process is minimal during the first three months and requires approximately 12 months for total resorption^[^24-26^]^.

## Approved BSR

As of 2023, six BRSs have European approval: Absorb, ART Pure, DESolve NXT, Magmaris, Fantom, and MeRes100. However, only Absorb has received FDA approval^[^18,24,27^]^. Four of these six scaffolds were PLLA-based, the most commonly used stent backbone. A summary of the advantages and disadvantages of currently approved BSR is presented in Table 1, highlighting their structural characteristics, clinical benefits, and associated limitations.

**Table 1:** Pros and cons of currently approved bioresorbable scaffolds. Each scaffold varies in material composition, drug elution, resorption time, and clinical performance, influencing its benefits and limitations in clinical use

Bioresorbable scaffold	Material	Drug elution	Strut thickness (μm)	Resorption time	Pros	Cons
**Absorb BVS** (Abbott)	PLLA	Everolimus	156	24–36 months	First FDA-approved BRS; controlled drug elution; restores vessel vasomotion post-resorption	Thick struts increase thrombogenicity; higher risk of target lesion failure; withdrawn from the US market
**DESolve NXT** (Elixir Medical)	PLLA	Novolimus	120	24–36 months	Thinner struts than Absorb; self-correcting properties for minor malposition; allows controlled vessel expansion	Requires careful implantation to prevent malapposition
**ART Pure** (Arterial Remodelling Technologies)	PDLLA	None	170	12 months	Shorter degradation time; non-drug-eluting (potentially reducing long-term inflammation)	Thicker struts; lacks drug-eluting benefits
**Magmaris** (BIOTRONIK)	Magnesium alloy	Sirolimus	150	12 months	Higher radial strength; less acute recoil; high vascular compliance allowing single-step inflation	Requires careful implantation to ensure proper positioning
**Fantom** (REVA Medical)	Desaminotyrosine-based polymer (DAT)	Sirolimus	125	12 months	Lower thrombogenicity; faster resorption time	Shorter follow-up data available; needs further long-term studies
**MeRes100** (Meril Life Sciences)	PLLA	Sirolimus	180	24 months	Comparable to Absorb; potential improvement in radial strength	Thicker struts may affect endothelialization

As previously stated, Absorb BVS (Abbott Vascular, Santa Clara, CA, USA) was the first BRS to be released. It is a PLLA-based everolimus-eluting BRS with a strut thickness of 156 μm. Absorb BVS has a coating thickness of 2–4 μm and a biosorption time of 24–36 months. The everolimus release mechanism in this model occurred purely by diffusion, with the coating material controlling its release^[^18,24,27,28^]^.

DESolve NXT (Elixir Medical, Sunnyvale, CA, USA) is another PLLA-based BRS that releases novalimus. It had a thinner strut thickness of 120 μm (the second generation, whereas the first generation had a standard thickness of 150 μm) and a coating thickness of <3 μm. DESolve has a similar resorption time of 24–36 months and first obtained its CE mark in 2014^[^18,24^].^ DESolve excelled over Absorb because it could self-correct itself in case of a minor malposition. It also has elasticity that permits intravascular expansion^[^24,27,29^]^.

**ART Pure (Arterial Remodelling Technologies, Paris, France)** is a PDLLA-based non-drug-eluting BRS with a thicker strut—170 μm. It has a short resorption time of approximately 12 months and was first given the CE mark in 2015^[^18,24^]^.

**Magmaris (BIOTRONIK AG, Buelach, Switzerland)** is a magnesium-based sirolimus-releasing BRS with a 150 Î 1/4 m strut thickness. Magmaris has a coating thickness of only 1 μm and bioresorption time of 12 months^[^18,24,27^].^ Magnesium offers magmaris with good radial strength, less acute recoil, and high vascular compliance, which allows it to be inflated using a single-step technique. A CE mark was obtained in 2016^[^18^]^. Magnesium-based BRS, such as Magmaris, allows lower thrombogenicity and higher strength (and radial strength) than regular PLLA BRS^[^24,27,30^]^.

**Fantom (REVA Medical, CA, USA)** is a desaminotyrosine-based (DAT)-based sirolimus-releasing BRS. It has a thin strut coating with a thickness of 125 μm and a short bioresorption time of 12 months^[^18,24,27^]^. A CE mark was obtained in 2017^[^18^]^.

**MeRes100 (Meril Life Sciences, Gujarat, India)** is a PLLA-based sirolimus-releasing BRS. It has a strut thickness of 180 μm and a resorption time of 24 months, and its CE mark was first obtained in 2019^[^18,24,27^]^.

## Absorb BVS US market withdrawal and the ABSORB III trial

Although the Absorb BVS 1.1 scaffold was the first to obtain the CE mark in 2011 and FDA approval in 2016, it was subsequently withdrawn from the US market a year later, in late 2017, due to lack of demand^[^31^]^ and concerns regarding possible short-to medium-term concerns regarding scaffold thrombosis and target lesion failure^[^21,32^]^. These concerns were further consolidated with the release of ABSORB III findings, suggesting a significantly higher incidence of target failure at 12 months post-stenting compared to DESs (7.8% vs. 6.1% for DES^[^33^]^ and at 3 years: − 13.4% vs. 10.4% for DES)^[^34^]^. Some proposed mechanisms for such findings include thicker scaffold struts that prolong stent endothelialization and enhance thrombogenicity^[^35^].^ Another possible cause is operator-dependent malapposition of the scaffold, which can prevent complete scaffold resorption^[^36^]^. Some researchers have even suggested that absorptive BVS has endogenous unexplained prothrombogenic properties^[^37^]^. Although the observed stent thrombosis was not associated with increased mortality, the withdrawal of Absorb stigmatized all BRSs.

## Adverse events associated with BRS

Although the incidence and mechanisms of BRS-related complications are still being studied, possible complications are associated with different clinical and procedural factors. The most common complications associated with BRS are stent thrombosis and restenosis. For example, acute and subacute stent thrombosis up to 30 days postimplantation is commonly associated with stent malapposition, incomplete lesion coverage, and poor implantation techniques (e.g., underemployment, stent fracture during implantation, or acute stent collapse and under expansion). In contrast, late-and very late-onset stent restenosis— > 1-month post-implantation—is often attributed to stent malapposition, endothelial inflammatory changes (e.g., neointimal proliferation), late scaffold fracture, uncovered stent struts, delayed stent resorption (> 3-4 years of structural retention), and vascular neoatherosclerosis^[^7,38,39^]^. These findings might be attributed to the inherent characteristics of bioresorbable stents, such as their weaker radial strength and gradual resorption^[^38^]^.

## Factors associated with higher risk of scaffold-related adverse events

Although trials are still being conducted to improve the BRS platform characteristics, the tensile strength and elasticity of newer models are still far lower tensile strength and elasticity when compared to those of metallic stents. For example, cobalt–chromium metallic stents (e.g., Xience) have a tensile strength of 1449 Mpa (vs. 220–330 Mpa for magnesium BRS) and tensile elasticity of 210–235 Gpa (vs. 45–55 Gpa for magnesium BRS). This means the currently used BRS models are 5- to 7-folds weaker and less elastic than regular metallic stents.

After identifying cases of stent thrombosis, prioritizing the identification of patients at higher risk for these complications is essential. A pooled analysis of the ABSORB EXTEND trial of almost 2000 patients^[^40^]^ aimed to identify the possible risk factors associated with a higher risk of cardiovascular complications. Regarding short-term (30-day) complications, female sex (HR = 3.6), prior myocardial infarction, prior PCI, residual stenosis >15%, and scaffold/reference vessel diameter ratio >1.25 were independently associated with short-term risk of cardiovascular complications. In contrast, patients with initial acute coronary syndrome were found to have the highest risk of long-term complications (HR = 2.79), followed by those with residual stenosis >15%, scaffold/reference diameter ratio >1.25, and dyslipidemia. Intravascular imaging was protective against long-term complications (HR = 0.13). Similar findings have also been reported in later studies, indicating that the optimum selection of BRS candidates is vital to prevent both short- and long-term complications^[^41,42^]^.

## Optimizing the use of BRS

**Figure 1.**
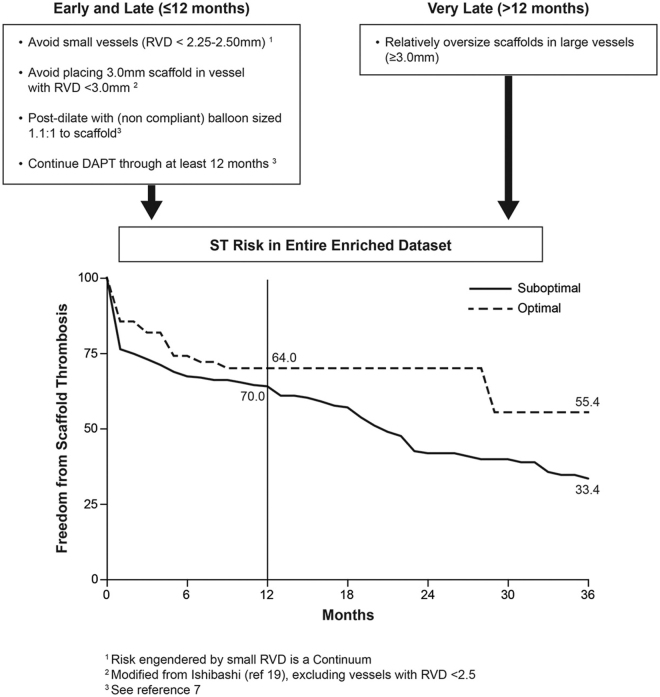
Factors to consider in order to minimize the risk of thrombosis of the bioresorbable scaffold, as suggested by Ellis et al.^[^39^]^ (reference 19 = ^[^44^]^ and reference 7 = ^[^45^]^) (ST: Stent thrombosis; RVD: Reference vessel diameter).

Although numerous trials are ongoing to overcome the adverse effects of different types of BRS, it is still important to ensure an optimum stent use strategy by developing newer models or using newer manufacturing technologies. Multiple studies^[^18,32,38,39,43^]^, as depicted in Fig. 1 ^[^39^]^ (In the figure reference 19 = ^[^44^]^ and refrence 7 = ^[^45^]^), have suggested factors to consider to optimize the use of BRS in clinical practice. These recommendations include the following.

**1) Proper patient selection**: This includes using the BRS in patients with expected long life expectancy and avoiding it in patients with saphenous venous graft thrombus, poor medical compliance, ST-elevation MI, severe vascular calcification, cardiogenic shock, or heart failure.

**2) Using an adequately sized scaffold**: Two factors should be considered when choosing the correct stent and vessel size: (1) optimum scaffold sizing based on the affected vessel diameter should be attempted, and (2) choosing the optimum vessel size for BRS implantation should be carefully considered (regardless of the scaffold size used). For example, stents smaller than the affected vessel should be avoided to prevent atherosclerotic plaque progression. In contrast, larger stents should be avoided because of the risks of strut overcrowding and reduced scaffold effectiveness. In addition, vessels with diameters less than 2.25 mm and more significant than 3.75 mm should be avoided to prevent scaffold thrombosis and stent dislocation, respectively.

**3) Adequate pre-implantation balloon dilatation**: Procedural considerations are critical to avoid early thrombosis. Optimum pre-implantation balloon dilatation involves using an inflation balloon to achieve minimum residual stenosis of <30%.

**4) Adequate post-implantation balloon dilatation**: With a balloon of the same size as the scaffold, the implantation result should not be controlled by intravascular imaging to document good apposition, less eccentricity, and residual stenosis of <20%.

## ABSORB IV results and BRS

With the release of the 5-year outcome results in 2023, the most extended follow-up in a BRS clinical trial to date, the ABSORB IV trial,^[^46^]^ we finally have solid information on the long-term efficacy and the incidence of very late complications of BRS, mainly Absorb BVS. The trial, performed on 2600 patients, reported that (1) only target lesion failure was higher in the BVS arm with a 3% absolute difference; (2) other complications, including major cardiovascular events, recurrent angina, and stent/scaffold thrombosis, were comparable with no significant differences, and the risk of stent thrombosis was only 0.8% higher with BVS only seen during the first 3 years post-implantation, and (3) no difference was observed in the quality of life of patients in both arms. According to ABSORB trials the results were more favorable when used in the vessel with a reference diameter of ≥2.25 mm^[^47^]^

## Advances and trials in the field of BRS

### 3D printing technology

Following the success of 3D printing technology in different medical fields, its incorporation into the field of BRS has been pursued^[^48-50^]^. The standard process of stent production, whether for BRS or regular stents, involves laser-beam machining of the primary stent material. However, such a technique may pose the risk of chemical and/or thermal stent issues. This is where the 3D printing came into the picture. 3D printing offers (1) a patient-customized stent model, (2) lower costs, and (3) no previously mentioned issues associated with regular stents^[^48^]^.

**Figure 2.**
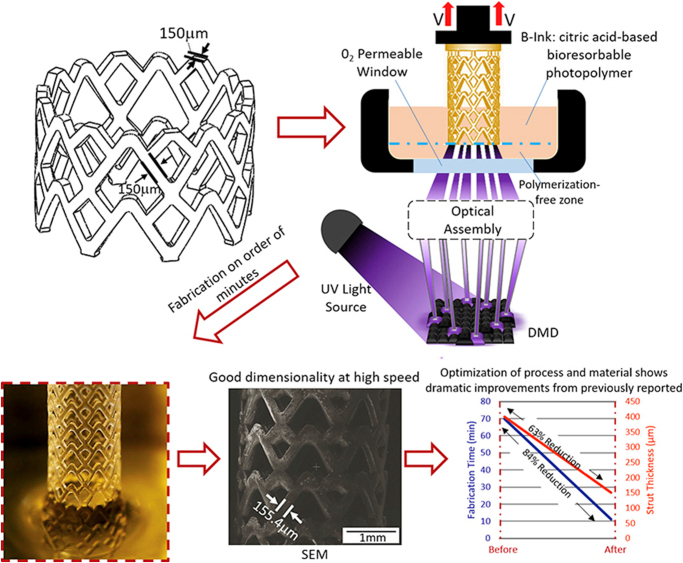
Showing the Ware *et al*’s model of (a) 3D-printed BRS design and dimensions, (b) μCLIP scaffold fabrication, and (c) Scanning electron microscope micrograph of 3D-printed BRS^[^48^].^ (μCLIP: micro-continuous liquid interface production; DMD: digital micro-mirror device; SEM: scanning electron microscope).

**Figure 3.**
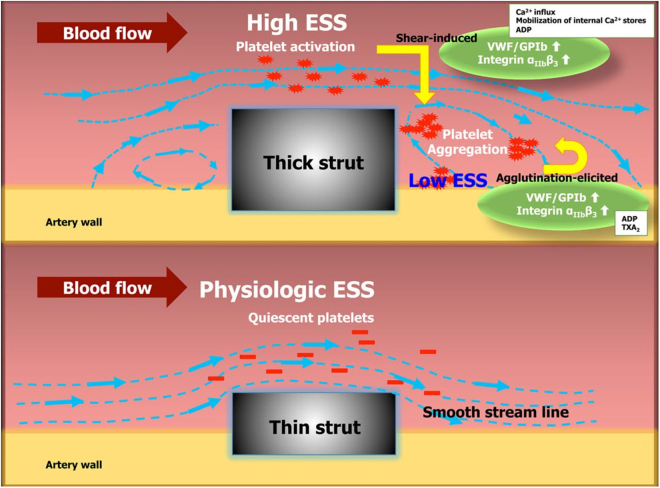
Mechanism of endothelial shear stress and vessel occlusion in thick bioresorbable scaffolds due to platelet aggregation^[^48^].^ (ESS: Endothelial shear stress; vWF: von Willebrand Factor; Gp: Membrane glycoprotein).

In 2018, Ware *et al*^[^48^]^ optimized the 3D printing of BRS using a high-resolution Micro-Continuous Liquid Interface Production (μCLIP) process (Fig. 2). They proposed that producing a 3D scaffold with adequate radial stiffness for regular BRS would require a strut thickness of 400 μm (versus 200 μm in Absorb BVS). Such thick scaffolds can reduce coronary blood flow in smaller vessels^[^48^]^ as depicted in Fig. 3. However, the authors’ proposed μCLIP calibration technique produced a BRS with comparable radial stiffness and suitable strut thickness (150 μm) in as low as 11.3 minutes, a significant improvement from previous trials^[^48^]^.

**Figure 4.**
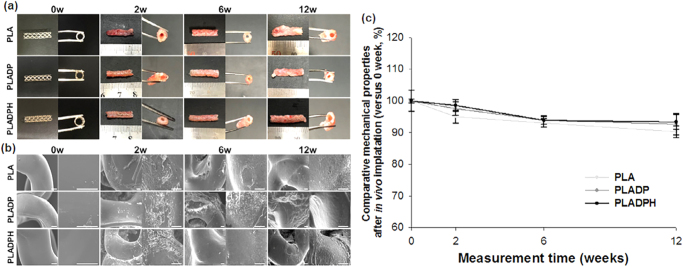
Changes in (a) gross appearance and (b) surface morphology of 3D-printed scaffold at 0 to 12 weeks of *in vivo* degradation, (c) is the measurement of radial force after *in vivo* implantation at each time point (0–12 weeks)^[^49^]^. (PLA: Heparinized poly l-lactic acid scaffolds; PLADPH: heparinized poly l-lactic acid scaffolds).

The second study by Lee *et al*^[^49^]^ proposed that using heparinized PLA 3D printed BRS could prevent restenosis and neointimal hyperplasia while providing good smooth muscle and endothelial cell modulation, as well as thromboresistant and blood compatibility properties (Fig. 4).

Another preclinical study by Shi *et al*^[^50^]^ used a novel sirolimus-eluting PLLA BRS, the AMSorb model (Beijing Advanced Medical Technologies, Beijing, China), and planted it into healthy porcine models. The authors demonstrated clinical safety, with the survival of all involved animals without stent thrombosis, fragmentation, or migration for >6 months. Although such a model is promising, the lack of a standard BRS control group (e.g., Absorb) leads to validity issues concerning the advantages of the listed 3D model. In addition, regular BRS models using the same material as AMSorb overgo total resorption in over three years, which would require a longer duration of follow-up^[^51^]^.

One recent preclinical study also used 3D-printed BRS and produced a 62 μm thick bare BRS (99 μm for drug-eluting stents) at an impressive production rate of one stent per minute. Despite such a short duration, stents demonstrated both safety and efficacy in preserving vessel function and preventing vessel inflammation over 28 days^[^52^]^.

Although 3D printing technology is still in its infancy when it comes to commercial use in BRS production, the rapid rate of improvement in the produced BRS models indicates a promising future for this technology to make BRS more commercially available at comparable quality.

### Newer bioresorbable scaffold generation models

Despite the approval of only six BRS models in Europe and only one in the United States, more and more attempts have been and are still being made to produce newer scaffold models that overcome the drawbacks of older generations.

**Figure 5.**
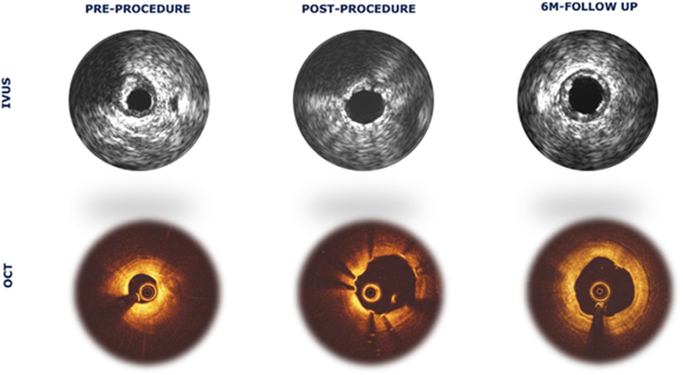
IVUS visualization (Upper row) and OCT visualization (Lower row) of the coronary vessel prior to the DREAM 3 G bioresorbable scaffold implantation procedure (pre-procedure), immediately after scaffold implantation (post-procedure), and 6 months after implantation^[^55^].^ (IVUS: Intravascular ultrasound; OCT: Optical coherence tomography).

- **FORTITUDE (Amaranth Medical, CA, USA**): FORTITUDE is a PLLA scaffold with a PDLLA coating. It is sirolimus-eluting (although some models do not) and has a strut thickness of 150 μm. Total resorption occurred within 10 months^[^32^]^. This model has a higher risk of vessel remodeling and luminal gain^[^53^]^.
- **XINSORB (HuaAn Biotechnology, China):** XINSORB is a sirolimus-eluting PLLA BRS with a 160 μm strut thickness^[^32^]^. The advantages of XINSORB in porcine studies include the inhibition of neointimal hyperplasia without inducing late-term device recoil (up to 180 days)^[^54^]^. These advantages are in addition to having similar efficacy to prior models, as well as a lack of major cardiovascular events and scaffold thrombosis^[^31^]^.
- **Firesorb (Microport, Shanghai, China**): Firesorb is a PLLA-based sirolimus-eluting stent with a PDLLA coating. It has two thicknesses: 125 μm (3 mm diameter) and 100 μm (2.5 mm diameter)^[^32^]^. This scaffold has a thinner strut thickness, and although it showed preliminary effectiveness over a 3-year period with a lack of scaffold thrombosis, myocardial infarction occurred in one patient (of 45 patients), requiring revascularization^[^31^]^.
- **DREAMS 3 G (Biotronik, Berlin, Germany**): DREAMS 3 G is a third-generation magnesium-based BRS. It has multiple customized strut thickness ranges, ranging from 99 μm (2.5 mm diameter; the thinnest to date) to 147 μm (4 mm diameter). The total resorption occurred within 12 months. The proposed advantages include a more extensive size range, increased X-ray visibility, and thinner struts. A thinner scaffold strut leads to 1) fewer complications and 2) better scaffold implantation and healing. Figure 5, presented by Haude *et al*, shows good scaffold opposition as well as non-visualization of the BRS at six months using Optical Coherence Tomography (OCT), with the scaffold only being detected on intravascular ultrasound^[^55^]^.

## Actionable Recommendations

Considering all the points discussed above, physicians need to (1) carefully select patients eligible for BRS insertion based on different individual factors, (2) properly select the appropriate BRS model, and (3) consider newer BRS production techniques (e.g., 3D printing) as well as newer yet-to-be-approved models. Choosing the optimum BRS model for the patient could minimize the risk of target lesion failure and scaffold-related short- and long-term complications as much as possible.

## Discussion and future directions

Optimal candidates for BRS intervention are crucial for ensuring better clinical outcomes. Factors influencing the selection process should be specified, including vessel size, lesion type, and patient comorbidities that may impact scaffold performance. For instance, patients with smaller vessel diameters or complex lesion morphologies might present heightened risks for scaffold thrombosis. Recent studies have begun to explore validated risk stratification models aimed at predicting the likelihood of BRS failure based on these parameters^[^2^]^. The incorporation of such models could guide clinicians in identifying the most suitable candidates for BRS implantation and potentially mitigate issues observed in earlier devices like the Absorb BVS.

In the realm of technological advancement, the integration of 3D printing into the fabrication of BRS provides opportunities for customization and optimization. The development of personalized scaffolds tailored to individual anatomical needs has become more feasible, addressing the previous limitations of standardized, commercially available stents. Various materials, such as polylactic acid (PLA) and its composites, are currently being investigated for their biocompatibility and mechanical durability as 3D-printed scaffolds^[^56,57^]^. Moreover, novel approaches involve the incorporation of bioactive additives to enhance scaffold functionalities; for example, the use of hydroxyapatite in polylactic acid scaffolds has shown promise in promoting osteogenic differentiation and better tissue integration^[^58,59^]^.

Magnesium-based BRS, such as the DREAM 3 G scaffold, introduces an alternative to polymer-based options. Preliminary trial data suggest that magnesium scaffolds exhibit a favorable combination of biodegradation and mechanical support characteristics, which differ significantly from their polymeric counterparts. Unlike polymeric scaffolds, which may exhibit prolonged degradation periods and pose risks for late-stent thrombosis, magnesium scaffolds have been demonstrated to degrade rapidly while maintaining adequate structural integrity during the healing process^[^60^]^. Their comparative advantages over polymeric scaffolds include improved flexibility and a potentially lower restenosis rate due to their unique degradation profiles, which minimize chronic foreign body response^[^61^]^. Future investigations should focus on addressing the specific safety and mechanical performance metrics of magnesium scaffolds in clinical settings as they evolve.

Bioresorbable scaffolds were first developed to overcome the disadvantages of permanent metallic stents, such as stent thrombosis or loss of vessel vasomotor capabilities. However, surprisingly, they showed a high incidence of scaffold thrombosis or target lesion failure, with even higher failure rates than permanent stents^[^33,34^]^. These findings have raised concerns regarding the use of bioresorbable scaffolds, with Absorb BVS being withdrawn from the US market. However, ongoing efforts have been made to (1) optimize BRS regardless of the implantation technique or the BRS models used^[^31,32,48-50,53-55^]^ and (2) upscale the trial cohort size and follow-up duration^[^48^]^. Findings from the ABSORB IV trial^[^46^]^ have revived the hope of using BRS, with similar long-term (>3 years) clinical outcomes for both BVS and permanent stents. Having a large-scale trial design similar to ABSORB IV^[^46^]^ for second-generation or even newer BRS models would be the arbitrator for future BRS in clinical settings.

## Conclusion

BRS have emerged as a promising alternative to traditional metallic stents, offering benefits such as restored vessel vasomotion and reduced long-term complications. Despite initial setbacks, including higher rates of thrombosis and restenosis, advancements in BRS design, manufacturing technologies such as 3D printing, and newer models such as DREAMS 3 G and XINSORB have demonstrated significant potential. Careful patient selection and adherence to optimized implantation protocols are pivotal. Continued research and large-scale trials are essential to establish BRS as a reliable coronary artery disease management option.

## Data Availability

Data are not publicly available.

## Research Registration Unique Identifying Number (UIN)

The study is a review article, so it does not require a Research Registration Unique Identifying Number (UIN).
